# High Electronically Conductive Tungsten Phosphate Glass-Ceramics

**DOI:** 10.3390/nano10122515

**Published:** 2020-12-15

**Authors:** Sanja Renka, Teodoro Klaser, Sanja Burazer, Petr Mošner, Petr Kalenda, Ana Šantić, Andrea Moguš-Milanković

**Affiliations:** 1 Division of Materials Chemistry, Ruđer Bošković Institute, 10000 Zagreb, Croatia; sanja.renka@irb.hr (S.R.); Sanja.Burazer@irb.hr (S.B.); asantic@irb.hr (A.Š.); 2 Department of Physics, Faculty of Science, University of Zagreb, 10000 Zagreb, Croatia; tklaser@phy.hr; 3 Department of General and Inorganic Chemistry, Faculty of Chemical Technology, University of Pardubice, 53210 Pardubice, Czech Republic; Petr.Mosner@upce.cz (P.M.); Petr.Kalenda2@upce.cz (P.K.)

**Keywords:** binary tungsten phosphate glass-ceramics, crystallization, electronic transport, W_2_O_3_(PO_4_)_2_ phase, semiconducting WO_3_ phase

## Abstract

High electronically conductive tungsten phosphate glass-ceramics have been prepared by the controlled crystallization of binary 60WO_3_–40P_2_O_5_ glass in the temperature range from 700 to 935 °C and for 1 to 24 h. The substantial increase in the conductivity for four orders of magnitude is a result of the formation of electronically conductive W_2_O_3_(PO_4_)_2_ and WO_3_ phases. At low crystallization temperature the dominant W_2_O_3_(PO_4_)_2_ phase is created, whereas at 935 °C for 24 h the formation of semiconducting WO_3_ crystallites of an average size of 80 nm enhances the conductivity to the highest value of 1.64 × 10^−4^ (Ω cm)^–1^ at 30 °C. The course of the crystallization and its impact on this exceptionally high electronic transport of binary tungsten phosphate glass-ceramics has been discussed in detail. Since such highly electronically conductive WO_3_-based glass-ceramics have a great potential as cathode/anode materials in solid state batteries and as electrocatalysts in fuel cells, it is of interest to provide a novel insight into the improvement of their electrical properties.

## 1. Introduction

The interest in glass-ceramics has grown exponentially in the recent years due to the novel and useful physical properties that can be obtained by controlling the crystallization process in the glass. Glass-ceramics contain at least one type of functional crystalline phase and a residual glass [1]. The transformation from glass to glass-ceramics takes place by a controlled process of nucleation and crystals growth giving rise to nano- and micro-structures [2]. The physical properties of glass-ceramics are strongly dependent on the size, type, morphology, and distribution of crystalline phases inside the glass matrix, all of which can be tuned by changing the crystallization conditions. For this reason, many functional glass-ceramics with improved mechanical, electrical, magnetic, and optical properties have been developed through the controlled glass crystallization. In particular, a significant scientific effort has been made to develop glass-ceramics which are applicable in the field of electronics and solid-state batteries as electrolytes or cathode/anode materials. However, ionic as well as electronic transport through the glass-ceramic material is by no means simple. Both conduction mechanisms depend on various structural and morphological characteristics of the material. For example, a study by Schirmeisen et al. [3] on lithium-ion conducting silicate glass-ceramics revealed a pivotal role of the interfacial regions that act as local electrical short circuits leading to an increase of the ionic conductivity of nanostructured glass-ceramics as compared to the corresponding glass.

Likewise, the electronically conductive oxide glasses containing transition metal oxides such as WO_3_, MoO_3_, V_2_O_5,_ and Fe_2_O_3_ appear to be versatile materials whose transport properties can be readily tailored by crystallization. In these glasses, electronic conduction occurs by the electron transfer between transition metal ions in different valence states following the small polaron hopping mechanism [4,5,6,7,8,9,10]. Expectedly, the crystallization strongly influences the formation of polarons and course of their transport. For instance, Garbarczyk and coworkers performed a series of studies on V_2_O_5_-P_2_O_5_-based glass-ceramics [11,12,13,14,15,16] and demonstrated that the polaronic transport can be greatly enhanced by the formation of interfacial regions around nano-crystalline grains. The authors postulated that these interfacial regions have a higher fraction of V^4+^-V^5+^ pairs and form an extensive network of electronic conduction paths which increases polaronic conductivity. On the other hand, our studies on the crystallization of Fe_2_O_3_-P_2_O_5_-based glasses [17,18] highlight the importance of nature of the phases that crystallize from the glass matrix. By widely varying the temperature and time of the heat-treatments, we were able to assign the changes in the conductivity to the interplay between amorphous glassy phase and crystalline mixed-valent iron and single-valent iron phosphate phases. In these systems, the conductivity decreases in the early stage of nano-crystallization due to the impoverishment of the continuous amorphous phase with Fe^2+^-Fe^3+^ pairs but increases with further formation of the interfacial region along well-defined interconnected crystalline grains.

Considering the crystallization processes in phosphate glasses containing WO_3_, there are many reports on tungsten oxide-based and tungsten fluorophosphate glass-ceramics, and their structural and optical properties [19,20,21,22]. Also, there are several reports on the transport mechanisms of the crystalline monophosphate tungsten bronze of the general formula (PO_2_)_4_(WO_3_)_2*m*_ [23,24,25] and tungsten oxide, WO_3_, crystals [26,27]. Recently, Moore et al. [28] revisited highly conductive WO_3_-TiO_2_-P_2_O_5_ glass-ceramic systems which were first reported by Aitken [26]. They showed that at low crystallization temperature tungsten oxide and titanium pyrophosphate are formed whereas at high temperature (PO_2_)_4_(WO_3_)_2*m*_ (*m* = 4–7) phases are created. Therefore, they concluded that the electrically conducting phases in these glass-ceramics are tungsten phosphates rather than tungsten suboxides reported hitherto.

Most recently, we investigated electrical transport in zinc phosphate glasses which contain alkali [29] and silver [30] oxides in combination with WO_3_. Our study showed that the exceptionally high electrical conductivity of WO_3_-rich glasses is a result of the formation of tungsten clusters of WO_6_ octahedra that facilitate the mobility of polarons. Further, our study on the controlled crystallization of 5Li_2_O-5ZnO-40P_2_O_5_-50WO_3_ (mol%) glass demonstrated that heat-treatments up to 800 °C induce crystallization of two polymorphs of W_2_O_3_(PO_4_)_2_ and a minor W_12_P_8_O_52_ phase [31]. The conductivity of obtained glass-ceramics is purely polaronic and close in value to that of the parent glass.

The present work aims to investigate the effect of the controlled crystallization on the electrical conductivity of binary 60WO_3_-40P_2_O_5_ (mol%) glass. The crystallization processes were induced by heat-treatments at different temperatures and times which enabled us to prepare glass-ceramics with various amounts of crystalline phases and comprehensively examine their role in electrical transport. According to our best knowledge, this is the first systematic study on the crystallization of binary tungsten phosphate glasses and its role in the enhancement of their transport properties.

## 2. Materials and Methods

The parent glass 60WO_3_-40P_2_O_5_ (mol%) was prepared by a conventional melt-quenching technique using an analytical grade of H_3_PO_4_ and WO_3_. The mixture of raw materials was firstly homogenized, calcined up to 600 °C, and held for 2 h in order to remove the water. The reaction mixture was then melted at 1350 °C in a platinum crucible with a lid and held at that temperature for 20 min. The melt was subsequently poured into a preheated graphite mold and annealed for 2 h at 515 °C (5 °C below *T*_g_). After cooling to room temperature (RT), the amorphous character of obtained dark blue glass was checked by X-ray diffraction analysis.

Based on the glass transition temperature (*T*_g_) and glass crystallization temperature (*T*_c_), heat-treatment temperatures for induced crystallization of glasses were selected as 700, 800, and 935 °C. The induced crystallization was performed in Nabertherm LHT 04/17 furnace with 10 °C/min heating rate and in steady air atmosphere. In the first series of experiments, the powder of 60WO_3_-40P_2_O_5_ glass was pressed in ~0.7 mm thick pellets and heat-treated for 1 and 6 h at 700 and 800 °C and 1, 12, and 24 h at 935 °C. The obtained glass-ceramics were mechanically strong and their color changed from blue to green with increasing crystallization time and temperature. In order to compare crystallization of the pelleted glass powder and bulk glass, the second set of experiments included the crystallization of 60WO_3_-40P_2_O_5_ glass in the form of ~1 mm thick discs for 1 and 12 h at 935 °C. However, the obtained glass-ceramics had much lower mechanical strength and were more prone to cracking in contrast to those prepared from pellets. The as-prepared glass-ceramics were labelled in accordance with their crystallization time and temperature where P and B stand for pellet and bulk crystallization, respectively. For example, P-935-1h glass-ceramic was prepared by heating glass pellet at 935 °C for 1 h.

X-ray diffraction data was collected at RT on Bruker D8 Discover diffractometer (Bruker AXS GmbH, Karlsruhe, Germany) equipped with LYNXEYE XE-T detector, in Bragg-Brentano geometry. Rietveld structure refinement was performed in HighScore Xpert Plus program 3.0 (Malvern Panalytical, Almelo, Netherlands). Vesta (free crystallographic software available online : https://jp-minerals.org/vesta/en/download.html) was used for crystal structure visualization [32]. Refinement was carried out by using the split-type pseudo-Voigt profile function and the polynomial background model. Isotropic vibration modes were assumed for all atoms. During the refinement, a zero shift, scale factor, half-width parameters, asymmetry, and peak shape parameters were simultaneously refined. Microstructural information was also obtained in the course of Rietveld refinement with LaB_6_ used as instrumental broadening standard.

The microstructure and elemental analysis of the prepared glass-ceramics were studied by field emission scanning electron microscopy FE-SEM JSM 7000 (JEOL, Welwyn Garden City, UK) equipped with the Oxford Instruments EDS/INCA 350 energy dispersive X-ray analyzer (EDS).

The density of the selected glass-ceramic samples, P-935-1h and P-935-24h, was determined at RT by the Archimedes method. Deionized water was used as buoyancy liquid. Measurements were done on three samples of both glass-ceramics and the average density for each glass-ceramic is reported. The random error in the density values was found to be ±1%. The relative density was estimated by comparing the measured density with the theoretical one calculated by the rule of mixtures considering 5.32 g/cm^3^ and 7.29 g/cm^3^ as the theoretical densities of W_2_O_3_(PO_4_)_2_ and WO_3_ phases, respectively.

For the electrical measurements, gold electrodes (3.8 mm diameter) were sputtered on both surfaces of the crystallized samples using the Sputter Coater SC7620 (West Sussex, UK). The samples were subsequently placed between two brass electrodes and the complex impedance was measured using an impedance analyzer (Novocontrol Alpha-AN Dielectric Spectrometer, Hundsangen, Germany) in a wide frequency (0.01 Hz–10^6^ Hz) and temperature (−30 °C to 240 °C) range. The temperature was controlled to an accuracy of ±0.2 °C.

## 3. Results and Discussion

### 3.1. Crystallization

According to the DTA results reported in the previous papers [29,30,33] the temperatures of heat treatments of binary 60WO_3_-40P_2_O_5_ glass were chosen to be in the temperature region above glass transition temperature (*T*_g_ = 520 °C) at 700 °C and 800 °C for 1 and 6 h and at crystallization temperature *T*_c_ = 935 °C for 1, 12 and 24 h. Additionally, samples in the form of discs were heat-treated at 935 °C for 1 and 12 h as described in the experimental section.

### 3.2. Structural Characterization

**Figure 1:**
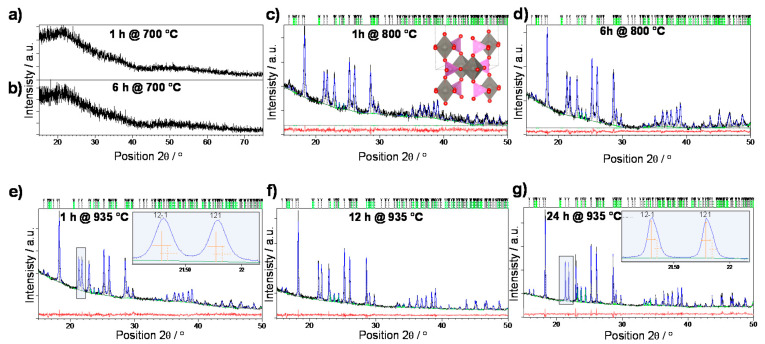
Rietveld refinements of glass pellet samples crystallized for (**a**) 1 h at 700 °C, (**b**) 6 h at 700 °C, (**c**) 1 h at 800 °C, (**d**) 6 h at 800 °C, (**e**) 1 h at 935 °C, (**f**) 12 h at 935 °C and (**g**) 24 h at 935 °C. Experimental data are given by black line, the calculated pattern is shown in blue, while the red line represents the difference curve. Grey vertical marks show the positions of diffraction lines belonging to W_2_O_3_(PO_4_)_2_ phase while the positions of WO_3_ lines are given as green vertical marks. Inset in (**c**) shows the structure of dominant phase, W_2_O_3_(PO_4_)_2_; WO_6_ octahedra are shown in grey while PO_4_ tetrahedra are pink. The insets in (**e**,**g**) demonstrate the differences in the integral line breadths caused by prolonged crystallization period.

The temperature-induced evolution of crystalline phases was investigated by the X-ray powder diffraction (XRPD). Figure 1 shows the results of Rietveld refinement analysis for glass pellets heat-treated at 700, 800, and 935 °C for the time ranging from 1 h to 24 h.

A wide scattering halo and absence of diffraction lines (Figure 1a,b) indicate that heat treatment at 700 °C, regardless of the heat treatment time, does not induce any crystallization processes and that the P-700 samples are characterized by an amorphous short-range ordering.

Heat treatment at 800 °C for 1 h represents the onset of crystallization with the appearance of diffraction lines corresponding to W_2_O_3_(PO_4_)_2_ (97.5 *wt.* %) and WO_3_ (2.5 *wt.* %) phases. The W_2_O_3_(PO_4_)_2_ phase crystallizes in the monoclinic system, P2_1_/*m* space group, with the refined unit-cell parameters: *a* = 7.796(6) Å, *b* = 12.526(5) Å, *c* = 7.777(6) Å, β=92.95(2)° similar to those reported by Kierkegaard and Asbrink (24072-ICSD) [34]. The structure consists of W_2_O_3_ dimer units (two WO_6_ octahedra connected in the corner shared manner) surrounded by PO_4_ tetrahedra (inset in Figure 1c). Each PO_4_ unit links together four dimer units, also *via* corner sharing, thus forming the 3D network. From the Figure 1c–g one can observe that monoclinic W_2_O_3_(PO_4_)_2_ remains the dominant crystalline phase in all the samples (positions of diffraction lines are given as grey vertical marks). However, the pronounced crystallization of WO_3_ (positions are given as green vertical marks) with a further increase of heat treatment time and temperature is also observed. The WO_3_ phase also crystallizes in monoclinic system, in space group P2_1_/*c* with the refined unit-cell parameters: *a* = 7.317(4) Å, *b* = 7.523(4) Å, *c* = 10.568(7) Å, β=133.05(2)° similar to those reported by Tanisaki (17003-ICSD) [35].

Quantitative compositions of the samples heat-treated at 700, 800 and 935 °C for different times of heat treatment are given in Table 1.

Based on the composition of samples given in Table 1, the general trend can be observed; the crystallization of WO_3_ phase becomes more pronounced with the increment of temperature but also, at both temperatures, prolongation in the heat treatment time results in the increase of WO_3_ phase fraction and the decrease in the amount of W_2_O_3_(PO_4_)_2_ phase.

Moreover, the X-ray line broadening analysis performed on the P-935-1h and P-935-24h samples revealed the pronounced growth of the diffraction domain sizes of the W_2_O_3_(PO_4_)_2_ phase with the prolonged time of heat treatment as seen from the evident decrease of integral line breadths for the sample annealed for 24 h compared to sample annealed for 1 h in the inset of Figure 1e,g. On the other hand, the WO_3_ phase does not show any significant difference in coherent diffraction domains sizes with prolonged heat treatment time; both samples crystallized for 1 h and 24 h contain crystallites with average sizes of ~80 nm.

**Figure 2:**
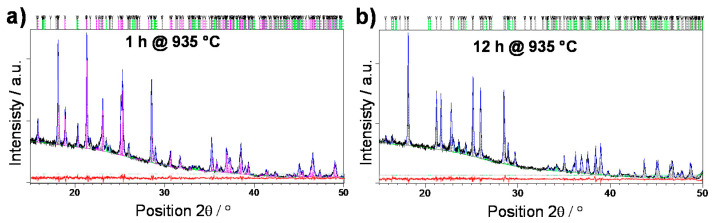
Rietveld refinements of the bulk glass samples crystallized for (**a**) 1 h and (**b**) 12 h at 935 °C. Experimental data are given by black line, the calculated pattern is shown in blue, while the red line represents the difference curve. Grey vertical marks show the positions of diffraction lines belonging to monoclinic W_2_O_3_(PO_4_)_2_ phase, magenta bars represent reflection of orthorhombic W_2_O_3_(PO_4_)_2_ phase, while the positions of WO_3_ lines are given as green vertical marks.

The crystallization from bulk samples have also been investigated by the means of quantitative Rietveld refinement as shown in Figure 2.

Interestingly, unlike the sample obtained from pellets, the sample obtained from bulk, when heat-treated at 935 °C for 1 h, shows the dominant presence of orthorhombic W_2_O_3_(PO_4_)_2_ phase as reported by Hanawa and Imoto (50742-ICSD) [36], accompanied by monoclinic W_2_O_3_(PO_4_)_2_ phase as well as small amount of WO_3_. With increase in heat treatment time, a polymorphic transition from orthorhombic to monoclinic phase W_2_O_3_(PO_4_)_2_ is observed, together with increase in the fraction of WO_3_ phase. The presence of the mixture of W_2_O_3_(PO_4_)_2_ polymorphs in B-935-1h indicates the difference in crystallization processes which occur within the bulk and surface of the glass. It seems that orthorhombic W_2_O_3_(PO_4_)_2_ starts to crystallize from the bulk and as crystallization proceeds it transforms to a monoclinic phase. This is in accordance with our study of crystallization of 5Li_2_O-5ZnO-40P_2_O_5_-50WO_3_ (mol%) glass where the heat treatments of the glass in the form of disc at 800 °C for 1 and 6 h also resulted in glass-ceramics containing both W_2_O_3_(PO_4_)_2_ polymorphs [31].

#### SEM Analysis

**Figure 3:**
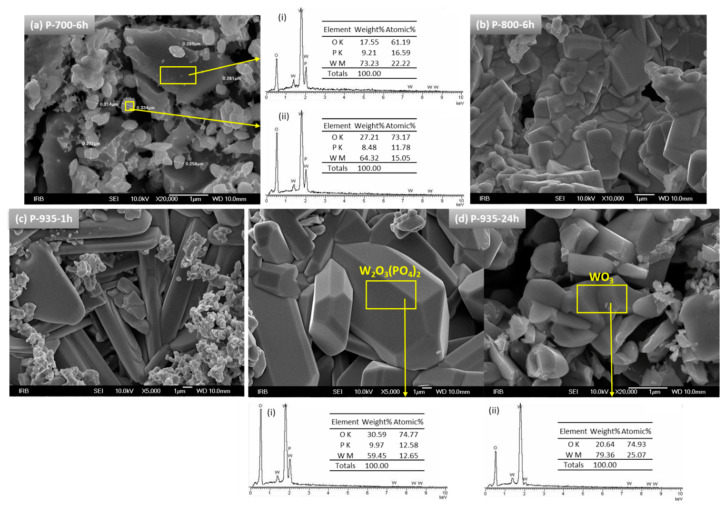
SEM micrographs of glass-ceramics prepared at (**a**) 700 °C for 6 h, (**b**) 800 °C for 6 h, (**c**) 935 °C for 1 h, and (**d**) 935 °C for 24 h. EDS spectra and tables with chemical composition from selected areas of the samples are shown in the insets (**i**) and (**ii**).

The insight into the microstructural features of glass-ceramics and crystalline grains was gained using scanning electron microscopy (SEM). Figure 3 exhibits the SEM micrographs of tungsten phosphate glass-ceramics obtained at various temperatures and for different times.

From Figure 3a, it can be seen that the P-700-6h sample contains grains of different sizes with no defined morphology. The EDS analysis shows that the chemical composition of the large grains corresponds to that of the parent glass, whereas smaller particles having relatively narrow size distribution of ~0.30 μm are stoichiometrically close to W_2_O_3_(PO_4_)_2_ phase, see tables in the upper-middle panel in Figure 3. Considering that the PXRD data do not show any diffraction lines it can be assumed that the small grains are amorphous, and, in fact, they act as precursors for further crystallization of W_2_O_3_(PO_4_)_2_ crystals.

At higher heat treatment temperature, namely 800 °C for 6 h, the microstructure shows better-defined grains with sharp edges and apexes which, according to PXRD, can be identified as dominant W_2_O_3_(PO_4_)_2_ phase. Also, large, elongated crystals, prismatic in shape, which appeared after crystallization at 935 °C for 1 h, are identified as W_2_O_3_(PO_4_)_2_ phase, see Figure 3c. When heat treatment at 935 °C is prolonged to 24 h, the W_2_O_3_(PO_4_)_2_ phase grows in size into large regular crystals of perfectly formed prismatic habit, Figure 3d. In accordance with PXRD results, this sample also contains sporadically distributed small grains of an irregular shape whose chemical composition matches WO_3_ phase, see EDS spectrum and table in the lower panel in Figure 3d. The size of WO_3_ grains is approximately ~1 μm, however, the X-ray line broadening analysis revealed that the crystallites within the grains have the average size of ~80 nm.

Noteworthy, the experimental densities of P-935-1h and P-935-24h glass-ceramics are significantly smaller than the corresponding theoretical values, P-935-1h: *ρ*_exp_ = 4.43 g/cm^3^ vs. *ρ*_theo_ = 5.37 g/cm^3^ and P-935-24h: *ρ*_exp_= 4.68 g/cm^3^ vs. *ρ*_theo_ = 5.58 g/cm^3^, giving the relative densities of 82% and 84%, respectively. This indicates high porosity of the obtained glass-ceramics which is in line with the SEM micrographs shown in Figure 3c,d.

### 3.3. Electrical Transport

**Figure 4:**
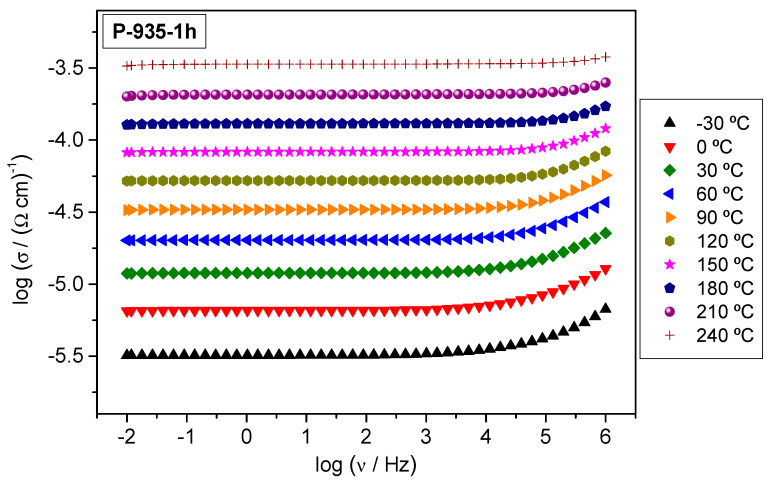
Conductivity spectra at different temperatures of glass-ceramics prepared at 935 °C for 1 h.

The conductivity spectra for P-935-1h glass-ceramics, as typical spectra for all samples in this study, is shown in Figure 4. Each isotherm exhibits a frequency-independent conductivity (DC conductivity) over a wide frequency range and a small frequency-dependent part at highest frequencies. These spectral features are characteristic for the electronically conducting materials and indicate fast non-localized electronic transport over a broad range of frequencies and temperatures. According to the PXRD analysis and SEM micrographs, samples heat-treated at 700 °C for 1 and 6 h show an amorphous structure with an indication of the stoichiometric rearrangement within the glassy phase whereas at higher crystallization temperatures, 800 and 935 °C, the crystallization processes rise creating two crystalline phases: W_2_O_3_(PO_4_)_2_ and WO_3_. Interestingly though, all samples, regardless of their structural and morphological properties, show frequency-independent conductivity over almost entire frequency span which signifies the absence of any blocking effects of the grain boundaries and (crystalline) grains to the conduction process. These effects usually manifest as a characteristic decrease of conductivity at lower frequencies and a corresponding low-frequency plateau. Therefore, it can be concluded that the electronic transport in all prepared glass-ceramics is continuous and uninterrupted.

**Figure 5:**
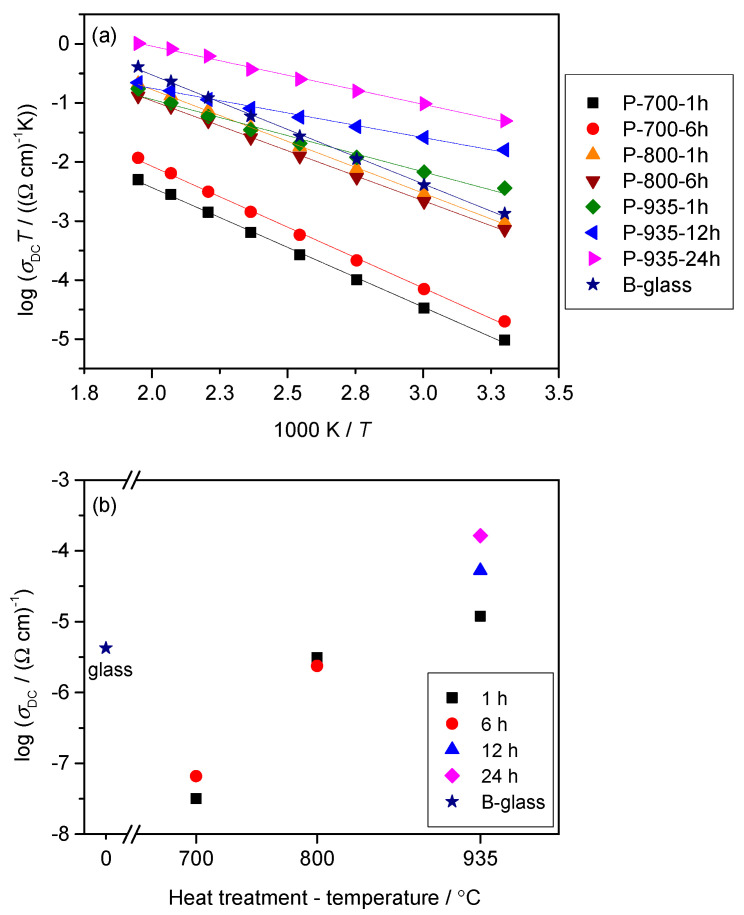
(**a**) Arrhenius plot of the parent 60WO_3_-40P_2_O_5_ glass and glass-ceramics prepared from pellets and (**b**) their DC conductivity, *σ*_DC_, at 30 °C as a function of crystallization temperature and time. The error bars are at most of the order of the symbol size.

For all samples, the DC conductivity exhibits Arrhenius temperature dependence and hence has characteristic activation energy, see Figure 5a. The activation energy for DC conductivity, *E*_DC_, for each sample was determined from the slope of log(*σ*_DC_*T*) vs. 1000/*T* using the equation *σ*_DC_*T* = *σ*_0_exp(−*E*_DC_/k_B_*T*), where *σ*_DC_ is the conductivity, *σ*_0_ is the pre-exponential factor, *E*_DC_ is the activation energy, k_B_ is the Boltzmann constant, and *T* is the temperature (K). The DC conductivity at 30 °C and determined activation energy, *E*_DC_, for the parent glass and all glass-ceramics are displayed in Table 2.

In Figure 5b, the dependence of *σ*_DC_ on the heat treatment conditions shows changes over four orders of magnitude which are correlated to the induced (micro)structural modifications.

As can be seen from Figure 5b, the heat treatments at 700 °C for 1 and 6 h result in nearly two orders of magnitude lower DC conductivity (*σ*_DC_ = 6.57 × 10^–8^ (Ω cm)^–1^ for P-700-6h at 30 °C), than the starting bulk glass (*σ*_DC_ = 4.26 × 10^–6^ (Ω cm)^–1^ at 30 °C). At this point, it is important to consider the structure and electrical transport within the parent bulk glass itself. Our recent studies [29,30] show that the exceptionally high DC conductivity of binary 60WO_3_-40P_2_O_5_ glass is a result of the formation of tungsten clusters of WO_6_ octahedra which facilitate the mobility of polarons *via* W^5+^–O–W^6+^–O–W^5+^ bonds. Remarkably, in tungsten phosphate glasses the most important factor of polaronic transport is found to be the structure of the glass network and not the fraction of reduced tungsten, W^5+^ ions [29,30]. Considering the high conductivity in starting 60WO_3_-40P_2_O_5_ bulk glass as a result of an easy motion of polarons through the tungsten clusters, it seems that the lower DC conductivity of P-700-1h and P-700-6h samples is related to a subtle rearrangement of the glass network during heating which accompanies breaking of the bonds in the tungsten clusters and formation of two fully amorphous but stoichiometrically different phases as shown by PXRD and SEM-EDS analysis, see Figure 1a,b and Figure 3a.

As the crystallization progresses and crystalline W_2_O_3_(PO_4_)_2_ and WO_3_ phases appear, the DC conductivity sharply increases to 3.11 × 10^–6^ (Ω cm)^–1^ and 2.38 × 10^–6^ (Ω cm)^–1^ at 30 °C for P-800-1h and P-800-6h samples, respectively. From the Rietveld analysis, it is evident that the W_2_O_3_(PO_4_)_2_ crystalline phase dominates in both samples having the relative proportion of 97.5 *wt.* % (P-800-1h) and 96.0 *wt.* % (P-800-6h), see Table 1. Also, the SEM micrograph of P-800-6h shows compact microstructure with well-connected crystalline grains which suggests that the observed increase in conductivity, in comparison to amorphous P-700-1h and P700-6h, is related to the fast electron transport within and/or along W_2_O_3_(PO_4_)_2_ grains. This agrees well with our recent investigation of the effect of the induced crystallization on the electrical conductivity of polaronic 5Li_2_O-5ZnO-40P_2_O_5_-50WO_3_ (mol%) glass [31] where we showed that crystallization of monoclinic and orthorhombic W_2_O_3_(PO_4_)_2_ as dominant phases in these glass-ceramics keeps the conductivity nearly the same as for the parent glass.

For glass-ceramics heat-treated at 935 °C for various times the DC conductivity increases further reaching the values of *σ*_DC_ = 1.20 × 10^–5^ (Ω cm)^–1^, *σ*_DC_ = 5.26 × 10^–5^ (Ω cm)^–1^, and *σ*_DC_ = 1.64 × 10^–4^ (Ω cm)^–1^ at 30 °C for P-935-1h, P-935-12h, and P-935-24h samples, respectively, see Table 2. According to the Rietveld analysis in these glass-ceramics, the relative amount of WO_3_ increases gradually from 4.3 *wt*. % for P-935-1h to 9.1 *wt*. % for P-935-12h and 18.0 *wt*. % for P-935-24h glass-ceramics, see Table 1. In line with the PXRD results, the SEM micrographs shown in Figure 3c, reveal long prismatic crystals that correspond to the W_2_O_3_(PO_4_)_2_ in the microstructure of P-935-1h whereas as crystallization proceeds with prolonged heating time to 24 h, along with the large W_2_O_3_(PO_4_)_2_ crystals, agglomerates of WO_3_ crystallites are evidenced, see Figure 3d. Here, it is worth noting that the X-ray line broadening analysis revealed that the size of WO_3_ crystallites within the grains does not change with the heat treatment time and remains app. ~80 nm in all glass-ceramics. Also, the relative density of P-935-1h and P-935-24h glass-ceramics is nearly the same (82–84%), indicating similar porosity and volume fraction of grain boundaries in both samples. Since the DC conductivity systematically increases with prolonged time of the heat treatment it can be concluded that the increase in the amount of nanostructured WO_3_ crystallites in these glass-ceramics is responsible for the observed rise of DC conductivity.

Let us now consider WO_3_ crystals_._ It is well known that WO_3_ is an n-type semiconductor with a wide band gap [37,38,39]. It undergoes several phase transitions as a function of temperature, with the monoclinic polymorph being stable at room temperature as also shown by our PXRD results. The electron concentration in WO_3_ is determined mainly by the concentration of stoichiometric defects such as oxygen vacancies which generate a new energy level below the conduction band. At the generated energy level, the active electrons can be transited to the upper energy levels. The conduction band generates free electrons and WO_3_ is thus electronically conductive [38,39]. Therefore, with increasing relative amount of WO_3_ crystallites in the P-935 glass-ceramics the concentration of free electrons also increases leading to the enhancement in DC conductivity. The obtained high DC conductivity, *σ*_DC_ = 1.64 × 10^–4^ (Ω cm)^–1^ at 30 °C for P-935-24h glass-ceramics, clearly reveals a rapid electron transport through WO_3_ crystallites as well as electronically conductive W_2_O_3_(PO_4_)_2_ crystals, resulting in high electronic conductivity.

**Figure 6:**
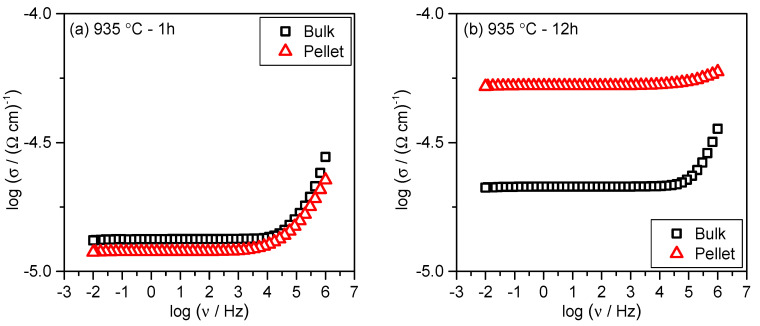
Comparison of the conductivity spectra measured at 30 °C for glass-ceramics prepared from the bulk glass (black squares) and pelleted glass powder (red triangles) at 935 °C for (**a**) 1 h and (**b**) 12 h.

Further, we analyze the electrical conductivity of the glass-ceramics prepared from the bulk and compare them with those prepared from the pellet. Figure 6 shows the frequency dependence of conductivity for the bulk and pellet samples heat-treated at 935 °C for 1 and 12 h. As can be seen from Figure 6a the electrical conductivity for both, P-935-1h and B-935-1h samples, shows the same values, *σ*_DC_ = 1.20 × 10^–5^ (Ω cm)^–1^ and *σ*_DC_ = 1.19 × 10^–5^ (Ω cm)^–1^ at 30 °C, respectively. The PXRD analysis shows that the bulk sample heat-treated for 1 h exhibits an orthorhombic W_2_O_3_(PO_4_)_2_ as a dominant phase along with monoclinic W_2_O_3_(PO_4_)_2_ phase and some traces of WO_3_. Structurally, these two W_2_O_3_(PO_4_)_2_ polymorphs do not differ substantially, so nearly identical values for the DC conductivity are expected. A slight decrease in the DC conductivity of bulk glass-ceramic crystallized for 12 h if compared with that prepared from the pellet, see Figure 6b, is a result of a slightly smaller amount of WO_3_ (8.2 *wt.* % for B-935-12h vs. 9.1 *wt.* % for P-935-12h) as well as a decrease in the crystals connectivity within the microstructure. In fact, glass-ceramics prepared from the bulk glass were very fragile and much less dense than their counterparts prepared from the pellets which implies that their conduction pathways are reduced and hence electron transport slower.

The analysis of the activation energy, *E*_DC_, for all samples shows an opposite trend to the DC conductivity, implying that the changes in microstructure have an impact on the activation energy. Also, a continuous decrease in the activation energy from 0.35 eV for P-700-1h to 0.17 eV for P-935-24h corresponds well to the previously reported data [40,41] for the n-type semiconductor behavior of WO_3_. Thus, the low activation energy of P-935 glass-ceramics acts as a facilitating factor in easy conductive pathways through the crystalline WO_3_ and W_2_O_3_(PO_4_)_2_ phases. On the other hand, the slightly higher activation energy for B-935-1h and B-935-12h glass-ceramics of 0.29 eV and 0.27 eV, mirrors a less compact microstructural network of the bulk samples.

## 4. Conclusions

A series of novel electronically conductive glass-ceramics were prepared by heat treatments of polaronic 60WO_3_-40P_2_O_5_ (mol%) glasses at different temperatures (700, 800, and 935 °C) and for various times (1, 6, 12, and 24 h). For glasses heat-treated at 700 °C for 1 and 6 h PXRD measurements show fully amorphous structure, however SEM-EDS micrographs reveal the presence of two types of grains; the large ones with the composition of the parent glass and the small ones which act as precursors for crystallization of W_2_O_3_(PO_4_)_2_. The heat treatments at higher temperatures induce the crystallization of W_2_O_3_(PO_4_)_2_ as a dominant phase and minor WO_3_ phase in all glass-ceramics. The amount of WO_3_ increases with increasing heat treatment temperature and time and reaches the highest value of 18.0 *wt.* % for the glass-ceramics prepared at 935 °C for 24 h. The electrical conductivity of prepared glass-ceramics is electronic in nature and shows a strong dependence on the evolution of crystalline phases. While the samples prepared at 700 °C have two orders of magnitude lower conductivity than the parent bulk glass due to the breakage of the tungsten clusters in glass network, the fast electronic transport within the W_2_O_3_(PO_4_)_2_ and WO_3_ crystalline grains makes glass-ceramics prepared at higher temperatures highly conductive. In particular, the increase of the amount of semiconducting WO_3_ crystallites of an average size of ~80 nm enhances the conduction of the glass-ceramics to the order of ~10^–4^ (Ω cm)^–1^ at 30 °C.

## Figures and Tables

**Table 1:** Quantitative composition of the samples heat-treated for 1 to 24 h at 700, 800, and 935 °C.

Sample	Heat Treatment Conditions		Composition (in *wt.* %)	
	*T*/°C	*t*/h	W_2_O_3_(PO_4_)_2_	WO_3_
P *-700-1h	700	1	-	-
P-700-6h	700	6	-	-
P-800-1h	800	1	97.5(1)	2.5(1)
P-800-6h	800	6	96.0(2)	4.0(1)
P-935-1h	935	1	95.7(2)	4.3(1)
P-935-12h	935	12	90.9(1)	9.1(1)
P-935-24h	935	24	82.0(1)	18.0(2)
B *-935-1h	935	1	76.1(2) (orthorombic) 19.1(2) (monoclinic)	4.8(2)
B-935-12h	935	12	91.8(1)	8.2(1)

* Labels P and B denote crystallization from pellet and bulk (discs), respectively.

**Table 2:** DC conductivity, *σ*_DC_, at 30 °C and activation energy, *E*_DC_, for parent 60WO_3_-40P_2_O_5_ bulk glass and prepared glass-ceramics.

Sample	*σ*_DC_/(Ω cm)^–1^ at 30 °C ± 1.0%	*E*_DC_/eV ± 1.0%
B-Glass	4.26 × 10^–6^	0.37
P-700-1h	3.18 × 10^–8^	0.35
P-700-6h	6.57 × 10^–8^	0.35
P-800-1h	3.11 × 10^–6^	0.30
P-800-6h	2.38 × 10^–6^	0.29
P-935-1h	1.20 × 10^–5^	0.18
P-935-12h	5.26 × 10^–5^	0.13
P-935-24h	1.64 × 10^–4^	0.17
B-935-1h	1.19 × 10^–5^	0.29
B-935-12h	2.14 × 10^–5^	0.27
